# Mitochondria: the cell powerhouse and nexus of stress

**DOI:** 10.3389/fphys.2013.00207

**Published:** 2013-08-07

**Authors:** Sabzali Javadov, Andrey V. Kuznetsov

**Affiliations:** ^1^Department of Physiology, School of Medicine, University of Puerto RicoSan Juan, Puerto Rico; ^2^Cardiac Surgery Research Laboratory, Department of Cardiac Surgery, Innsbruck Medical UniversityInnsbruck, Austria

Mitochondria, sub-cellular organelles originated from primary endosymbiosis, play a vital role in the energy metabolism of eukaryotic cells. Despite many aspects of ATP synthesis have been delineated, regulatory mechanisms responsible for energy synthesis and transfer still remain to be uncovered. In addition to production of energy, mitochondria play a crucial role in entire cell metabolism under both physiological and pathological conditions through their participation in cell death, ion homeostasis, reactive oxygen species (ROS) generation, redox signaling as well as various intracellular signaling pathways. Studies over the last 30 years provide strong evidence that mitochondria are the nexus of various stresses which initiate cell death. The Research Topic “Mitochondria: the cell powerhouse and nexus of stress” presents state-of-the-art studies in mitochondrial research performed by prominent experts from different laboratories around the world. All 13 original research and review articles presented in the topic are dedicated to several aspects of mitochondriology including morphology, dynamics, metabolism, function, and regulation of these organelles under normal and pathophysiological conditions.

It should be noted that despite many studies, numerous aspects of mitochondrial functioning including the precise mechanisms of protein synthesis in mitochondria remain unclear. In the study presented by Koc et al. (2013) two novel small subunit proteins, CHCHD1 and AURKAIP1, and the large subunit protein, CRIF1 were characterized as new members of the mammalian mitochondrial ribosome. Using siRNA knock-down studies the authors demonstrated crucial roles of these proteins in mitochondrial protein synthesis, and revealed their significant effects on the expression of mitochondrially encoded proteins. Also, interaction of mitochondria with cytoplasmic proteins plays a causative role in the regulation of energy metabolism and cell death. Recent studies revealed interaction between elements of cytoskeleton and mitochondria which may modulate energy synthesis and transfer (Rostovtseva and Bezrukov, 2008; Guzun et al., 2011). The review by Kuznetsov et al. (2013) summarizes studies on the possible role of the cytoskeletal protein β-tubulin II in the regulation of mitochondrial metabolism, respiratory function, and energy transfer. The authors suggest that interaction of β-tubulin II with mitochondria can participate in the coupling of ATP-ADP translocase (ANT), mitochondrial creatine kinase (MtCK), and voltage-dependent anion channel (VDAC), and the ANT-MtCK-VDAC complex is responsible for the efficient intracellular energy transfer via the phosphocreatine pathway. Kaambre et al. (2013) demonstrated the applicability of the metabolic control analysis as a promising method for quantification of the flux control exerted by different enzymatic steps in total metabolic network that can be applied to study mechanisms of energy metabolism in human breast and colorectal cancer cells. In addition to interaction with other proteins/organelles, mitochondria *per se* can undergo structural modifications known as mitochondrial dynamics which is associated with changes in their number, size, shape, and intracellular localization. Mitochondrial dynamics is regulated by the balanced action of various proteins responsible for fission and fusion. These processes play also a key role in Ca^2+^ regulations, as well as in biogenesis and the quality-control of mitochondria under normal physiological conditions. Enhanced fission of mitochondria is associated with mitochondrial fragmentation, which is early sign of apoptosis in various cell stresses, including cardiac diseases or ischemia-reperfusion injury although molecular mechanisms of cell death induced by alterations in mitochondrial dynamics remain poorly understood. In this respect, the study presented by Piquereau et al. (2013) discusses the possible role of mitochondrial dynamics in mitochondria-mediated cardiac dysfunction in ischemia/reperfusion and heart failure. The authors highlight an importance of mitochondrial dynamics in mitochondrial and cellular physiology.

It is known that a number of signaling molecules including cytoplasmic and/or mitochondrial nitric oxide (NO) and ROS participate in the regulation of mitochondrial metabolism. Synthesis of NO from its precursor, nitrite increases during hypoxia and enhanced NO through cGMP and can protect the heart from ischemia/reperfusion injury. However, the mechanisms of the synthesis of NO from nitrite in cardiomyocytes are still not fully understood. Dungel et al. (2013) demonstrated that mitochondria play a predominant role in nitrite reduction to NO, leading to enhanced cGMP synthesis in cardiomyocytes. Notably, mitochondrial ROS levels can be strongly regulated by several distinct ROS-detoxification mechanisms. Expression of the major ROS scavenger enzymes superoxide dismutase 2 (SOD2), catalase, or sestrins is shown to be regulated by Forkhead box O (FOXO) transcription factors. Hagenbuchner and Ausserlechner (2013) discuss balanced action between Bim, mitochondrial architecture, and ROS-detoxifying proteins that are regulated by FOXO. Importantly, ROS-mediated modulation of various intracellular signaling pathways can also be involved in the regulation of heart contractility by the cardiac renin-angiotensin II-aldosterone system (RAAS). Activation of RAAS enhances both cytoplasmic and mitochondrial ROS generation although the cause-effect relationship between these two sources for ROS remains to be elucidated. De Giusti et al. (2013) presented a comprehensive review on the relationship between chronic RAAS stimulation and mitochondrial ROS, and the possible role of sodium exchangers in RAAS-mediated cardiac hypertrophy. Consequently, pharmacological inhibition of mitochondrial ROS generation exerts a beneficial effect. Investigating ROS in skeletal muscles, La Guardia et al. (2013) demonstrated that oxidative damage induced by simvastatin in fibers from rat skeletal muscle is associated with the enhanced mitochondrial superoxide generation, whereas pretreatment with L-carnitine prevented the toxic effects of simvastatin. Interestingly, the study presented by Canzoniero et al. (2013) shows that neurons expressing large amounts of nNOS produce significantly less mitochondrial ROS in response to an excitotoxic challenge, thereby providing a potential mechanism for reduced cells vulnerability to the excitotoxicity in patients with Huntington's disease. It is well-known that a number of various mitochondrial diseases are associated with mutations, deletions, or deficiency of genes encoding mitochondrial proteins. Deficiency of mitochondrial phospholipid cardiolipin in humans known as Barth syndrome is caused due to absence of mitochondrial acyl-transferase, tafazzin that is essential for remodeling acyl chains of cardiolipin. Powers et al. (2013) demonstrated that diminished exercise capacity in tafazzin-knockdown mice can be explained by the respiratory dysfunction of mitochondria. A causative link between mitochondrial dysfunction and pathological disorders has been shown using different other models of diseases. For example, it has been shown that mitochondria dysfunction plays also an important role in developing of inflammation. Experiments using rat liver slices and isolated mitochondria demonstrated that inflammation induced by inflammatory mediators resulted in mitochondrial dysfunction due to secondary hypoxia (Weidinger et al., 2013). This study revealed hypoxia-induced attenuation of complexes I and II function in response to inflammation.

Accumulation of ROS along with Ca^2+^ overload can be considered as the major factors causing mitochondrial permeability transition (mPT) associated with depolarization of mitochondrial membrane, ATP depletion and opening of non-specific pathological mPT pores (mPTP). Irreversible high-conductance mPTP opening plays a critical role in mitochondria-mediated cell death. Although opening of mPTP is well-known phenomenon, the molecular identity of the pores is still elusive. Previous findings that the mPTP consists of VDAC and ANT were not proven by genetic studies when VDAC- or ANT-null mitochondria still exhibited a CsA-sensitive pore opening [reviewed in Bernardi (2013)]. Cyclophilin D (CyP-D) is the only defined component which plays an important regulatory role in pore opening. Interestingly, recent studies demonstrated that CyP-D binds the lateral stalk of the *F*_0_*F*_1_ ATP synthase and modulates its activity (Giorgio et al., 2009). Furthermore, the authors provided strong evidence that dimers of the *F*_0_*F*_1_ ATP synthase incorporated into lipid bilayers form Ca^2±^-activated channels with the key features of the mPTP (Bernardi, 2013; Giorgio et al., 2013). The review by Javadov and Kuznetsov (2013) summarizes and discusses the possible mechanisms of the activation of CyP-D to interact with a target protein in the inner mitochondrial membrane and initiate opening of the mPTP. Notably, physiological (low-conductance) mPTP opening can regulate mitochondrial Ca^2+^ homeostasis through modulation of Ca^2+^ efflux, and generate and convey calcium signals from one mitochondrion to another (reviewed in Brenner and Moulin, 2012). Indeed, the low-conductance mPTP is one of dozens of channels/exchangers which regulate ion homeostasis in mitochondria. In this aspect, Na^+^/H^+^ exchanger 1 (NHE1) is one of most interesting ion transporters recently found in the mitochondrial inner membrane (Javadov et al., 2011; Villa-Abrille et al., 2011). Although NHE1 is the main transporter of the plasma membrane regulating H^+^ concentration in cytoplasm, its role in mitochondrial physiology is still unknown. In this Topic, the potential role of mitochondrial NHE1 as a target to prevent cardiac diseases such as ischemia/reperfusion and heart failure was described by Alvarez and Villa-Abrille (2013).

In conclusion, this Frontiers Review Topic highlights several important aspects of mitochondrial research under normal and pathological/stressful conditions. This will hopefully be useful for further understanding of mitochondrial biology as well as molecular mechanisms of mitochondria-mediated cell dysfunction aiming to develop a number of new therapeutic approaches for the treatment of various human diseases by targeting mitochondria.
